# An Optical Natural Curiosity

**Published:** 1817-10

**Authors:** Thomas Renwick


*For the London Medical and Physical Journal.*

### *An Optical Natural Curiosity;* by THOMAS RENWICK, M.D.

THE notice you have taken in the Medical and Physical Journal for April last, of a curious optical phenomenon which has appeared at Liverpool, as well as the remarks upon the same subject in July, have not escaped my observation. I am sorry the public have been led to expect an account of this case sooner than it could with propriety be given.

My patient, Miss M'Avoy, who has been made an object of public curiosity by the powers she is supposed to possess of distinguishing colours, reading, &c. has been subject to repeated attacks of disease, which have occasioned great interruption to the examinations which were thought necessary

 before before her case could be presented to the public. The generality of persons who have visited her are satisfied that Miss M'Avoy really possesses the powers attributed to her. She has failed, however, in one or two examinations, when several respectable individuals of the profession were present, and they have generally expressed not only doubts, but a few have given a positive opinion, that Miss M'Avoy can see, and that the powers ascribed to her are deceptive. On the other hand, many gentlemen, both in and out of the profession, of equally respectable talents, have been convinced she does possess the powers ascribed to her. Whatever may hereafter be proved, it will be sufficient, until the case be published, to say, that Miss M'Avoy became blind on the 6th or 7th of June 1816, and that she continued so for several months. The symptoms which had occurred previous to that period were indicative that such an event might reasonably be expected. The pupil contracts and dilates, although not in the most perfect manner; but this was not perceived to be the case, until the middle of October, and is not a certain proof of Miss M'Avoy's possessing the power of vision. My own observations satisfy me, that, whether Miss M'Avoy can see or not, the modes adopted to blindfold her have been sufficient for that purpose, and that she has distinguished colours, read, and shown herself possessed of powers still more extraordinary. Miss M'Avoy, after distinguishing colours, reading, &c. often suddenly loses the power, and every thing appears black; the fingers become cold; and the power often returns as suddenly as it was lost, upon the fingers becoming warmer. In those trials where the eyes were uncovered, she has as frequently failed as when the eyes are covered.

I am of opinion that Miss M'Avoy does not see, yet I am not surprized that other persons who have not examined into her situation as much as I have done, should express doubts on the subject. If it should be proved, however, that Miss M'Avoy does see, I shall have no hesitation in retracting an opinion which has not been formed upon slight grounds, nor without the due investigation, a subject of this importance demands. In a medical point of view also, this case is very curious, and I hope it will be in the possession of the public in the course of the next month.

*Liverpool; August* 23, 1817.

